# Mesomere-derived glutamate decarboxylase-expressing blastocoelar mesenchyme cells of sea urchin larvae

**DOI:** 10.1242/bio.20136882

**Published:** 2013-12-11

**Authors:** Hideki Katow, Tomoko Katow, Kouki Abe, Shioh Ooka, Masato Kiyomoto, Gen Hamanaka

**Affiliations:** 1 Division of Developmental Biology, Research Center for Marine Biology, Tohoku University, Asamushi, Aomori 039-3501, Japan; 2 Marine and Coastal Research Center, Ochanomizu University, Tateyama, Chiba 294-0301, Japan; ‡ Present address: Nara Institute of Science and Technology, Laboratory of Neuronal Cell Morphogenesis, Graduate School of Biological Sciences, Ikoma 630-0192, Japan; § Present address: Tokyo University of Marine Science and Technology, Field Science Center, Tateyama Station (Banda), Chiba 294-0308, Japan

**Keywords:** Cell lineage, Mesomere, Mesenchyme, GAD cell, Chimera, Epithelial-to-mesenchymal transition, Sea urchin

## Abstract

The ontogenetic origin of blastocoelar glutamate decarboxylase (GAD)-expressing cells (GADCs) in larvae of the sea urchin *Hemicentrotus pulcherrimus* was elucidated. Whole-mount *in situ* hybridisation (WISH) detected transcription of the gene that encodes GAD in *H. pulcherrimus* (*Hp-gad*) in unfertilised eggs and all blastomeres in morulae. However, at and after the swimming blastula stage, the transcript accumulation was particularly prominent in clumps of ectodermal cells throughout the embryonic surface. During the gastrula stage, the transcripts also accumulated in the endomesoderm and certain blastocoelar cells. Consistent with the increasing number of *Hp-gad* transcribing cells, immunoblot analysis indicated that the relative abundance of Hp-Gad increased considerably from the early gastrula stage until the prism stage. The expression pattern of GADCs determined by immunohistochemistry was identical to the pattern of *Hp-gad* transcript accumulation determined using WISH. In early gastrulae, GADCs formed blastocoelar cell aggregates around the blastopore with primary mesenchyme cells. The increase in the number of blastocoelar GADCs was inversely proportional to the number of ectodermal GADCs ranging from a few percent of total GADCs in early gastrulae to 80% in late prism larvae; this depended on ingression of ectodermal GADCs into the blastocoel. Some of the blastocoelar GADCs were fluorescein-positive in the larvae that developed from the 16-cell stage chimeric embryos; these comprised fluorescein-labeled mesomeres and unlabelled macromeres and micromeres. Our finding indicates that some of the blastocoelar GADCs are derived from the mesomeres and thus they are the new group of mesenchyme cells, the tertiary mesenchyme cells.

## Introduction

The adult nervous system (ANS) of sea urchins is unique among the deuterostomes in being pentaradial; it comprises five radial nerves that are connected with axons of the circumoral nerve ring, which itself contains few nerve cell bodies. Thus, adult sea urchins have been regarded as having no brain (Peterson et al., 2000). On the other hand, the larval nervous system (LNS) retains the bilateral body plan of deuterostomes, with an apical ganglion that contains dozens of synaptotagmin-expressing serotonergic and non-serotonergic neurons on the dorso-frontal side of the larval mouth (Yaguchi and Katow, 2003; Katow et al., 2009; Elia et al., 2009; Hoekstra et al., 2012). The ontogenetic analysis of LNS has been fairly well documented, particularly for the serotonergic NS. The ANS is connected to the LNS at a node near the adult rudiment by synaptotagmin-expressing nerves (Katow et al., 2009). The serotonergic NS is present at a trace level in the adult rudiment of *Holopneustes purpurascens* for a short period during the early stage of metamorphosis, but is absent in adults (Katow et al., 2009). This is also seen in *Hemicentrotus pulcherrimus* (Katow et al., 2010). Thus, the ontogenetic analysis of the ANS had not advanced much since it was first described at the end of the 19th century (Bury, 1895). However, recently, in addition to synaptotagmin-expressing NS, γ-amino butyric acid (GABA)-ergic NS is detected distinctively in the primary podia and the epineural fold of the adult rudiment in *H. pulcherrimus* (H.K., unpublished). This suggests the presence of ontogenetic continuity from the LNS to the ANS in sea urchin, which enables analysis of the ontogeny of GABAergic NS from embryo to adult.

Larval swimming is regulated by at least three regulatory systems (RSs) of classic neurotransmitters in the sea urchin, *H. pulcherrimus*. These are the serotonergic (Yaguchi et al., 2000; Yaguchi and Katow, 2003; Katow et al., 2004; Katow et al., 2007), dopaminergic (Katow et al., 2010) and GABAergic (Katow et al., 2013) RSs. Among them, the serotonergic NS differentiates last in the apical ectoderm around the prism stage (Yaguchi et al., 2000; Byrne et al., 2007) by the mechanism that regulates ectodermal regionalisation (Yaguchi et al., 2011; Angerer et al., 2011; Yaguchi et al., 2012), and innervates the digestive organs, the circumoral ciliary bands (Katow et al., 2007; Katow et al., 2009; Katow et al., 2010) and the epaulets of plutei (Katow et al., 2013). However, the formation of synaptotagmin-expressing uncharacterised non-serotonergic pharyngeal neuron is reported in the endoderm of four-day post-fertilisation (-dpf) two-arm plutei (2aPlut) of *Strongylocentrotus purpuratus* (Wei et al., 2011). This implies that both the ectoderm and the endoderm participate in NS formation in the sea urchin embryo. The dopaminergic RS is the next latest system that appears; it is closely associated with the basal bodies of cilia from the unhatched motile blastula stage, and converges to the circumoral ciliary band by the two-dpf 2aPlut stage and the epaulets by the 30-dpf eight-arm pluteus stage (Katow et al., 2010).

The GABAergic NS is the first of the above three swimming RSs of the sea urchin embryo to appear; this was established by using reverse transcription PCR (RT-PCR) to determine, in fertilized eggs of *H. pulcherrimus*, the abundance of *Hp-gabarap* transcripts, which encode the GABA_A_ receptor-associated protein, and *Hp-GabrA* transcripts, which encode the GABA_A_ receptor (Katow et al., 2013). The GABA-expressing cells express glutamate decarboxylase (GAD) and, unlike the aforementioned two ectodermal RSs, are detected in the blastocoelar mesenchyme of 2aPlut. Later in development, some of the GAD-expressing cells (GADCs) form a stripe on the apical surface of four-dpf four-arm plutei (4aPlut) (Katow et al., 2013); this suggests the egression of GADCs from the blastocoel to the apical surface of larval ectoderm. There are two groups of mesenchyme cells in sea urchin embryo. The first comprises mesenchyme is the primary mesenchyme cells (PMCs) that differentiate into spicules exclusively in the blastocoel (Hörstadius, 1939; Okazaki, 1975) and can thus be ruled out as a possible ancestor of GADCs. The only mesenchyme cell group other than PMCs is the pluripotent secondary mesenchyme cells (SMCs) that migrate into the blastocoel. SMCs are formed in the vegetal ectoderm as early as the blastula stage, and differentiate into various cell types, such as coelomic pouch cells and circumesophageal muscle cells (Ishimoda-Takagi et al., 1984), serotonin receptor cells (SRCs) (Katow et al., 2004) and pigment cells that migrate to the apical surface of embryos (Calestani and Rogers, 2010; Lyons et al., 2012). This suggests that SMCs might be the ontogenetic precursors of GADCs. However, given that no SMC descendant has been reported to differentiate into GADCs, this study was aimed to elucidate the ontogenetic origin of GADCs.

## Materials and Methods

Sea urchins (*H. pulcherrimus* A. Agassiz) were collected around the Research Center for Marine Biology, Tohoku University, Japan. Gametes were obtained by intracoelomic injection of 0.5 mol l^−1^ KCl. Eggs were inseminated and incubated in filtered sea water (FSW) on a gyratory shaker or stirred gently with a propeller in plastic containers in an incubator at 15°C or 18°C until the appropriate developmental stages were reached. Larvae were fed *Chaetoceros calcitrans* (Nisshin Marine Tech. Ltd., Yokohama, Japan) from four days after fertilisation until the day described in the text.

### Whole-mount *in situ* hybridisation

Whole-mount *in situ* hybridisation (WISH) and whole-mount fluorescent *in situ* hybridisation (WFISH) were performed as described (Abe et al., 2013). Unfertilised eggs (Ufe), eggs 30 minutes post-fertilisation (Fe), 6-hour post-fertilisation (hpf) morulae (Morula), 15-hpf swimming blastulae (sBl), 17-hpf mesenchyme blastulae (mBl), 18-hpf early gastrulae (eG), 22-hpf late gastrulae (lG) and 24-hpf prism larvae (Prism) were fixed with 4% paraformaldehyde diluted in mixture of FSW (4% PFA), 10 mmol l^−1^ 3-morpholinopropanesulphonic acid (MOPS) buffer and 0.1% Tween 20 at ambient temperature (AT) for one hour.

A fragment of the gene that encodes Hp-Gad (527 bp; *Hp-gad*, AB713401) was isolated from sea urchin embryos using the following primers: *Hp-gad*-F, 5′-ACTACGCGCCAAAGACCTTC-3′; and *Hp-gad*-R, 5′-TCCACGTCTCTTGCATGCTG-3′. Sense and antisense RNA probes were synthesised using SP6 and T7 RNA polymerases and digoxygenin (Dig) RNA labelling mix (Roche, Basel, Switzerland). Before hybridisation, the above samples were treated with 2 µg ml^−1^ Proteinase K (Wako, Osaka, Japan) in MOPS buffer for 15 minutes at 37°C, washed twice with MOPS buffer, fixed with 4% paraformaldehyde diluted in MOPS buffer for 20 minutes, washed with MOPS buffer three times, and incubated with hybridisation buffer [70% formaldehyde, 0.1 mol l^−1^ MOPS (pH 7.0), 0.5 mol l^−1^ NaCl, 0.1% Tween 20, 1 mg ml^−1^ bovine serum albumin (BSA)] for three hours and hybridised with 0.7 ng µl^−1^ RNA probes at 45°C for seven days. After hybridisation, the samples were washed with the hybridisation buffer for six hours. After further washing with MOPS buffer five times, the blocking was conducted twice with 1% skim milk in MOPS buffer (blocking buffer) for 15 minutes and then in MOPS buffer containing 10% goat serum and 1 mg ml^−1^ BSA for 15 minutes at 37°C. Alkaline phosphatase (AP)-tagged goat anti-Dig antibody (Roche) was diluted in MOPS buffer containing 1% goat serum and 0.1 mg ml^−1^ BSA, and incubated overnight at AT. The samples were washed five times with MOPS buffer, which was replaced with fresh AP buffer for 30 minutes before determining the location of AP-tagged anti-Dig antibody location was detected by incubation in nitroblue tetrazolium/5-bromo-4-chloro-3-indolyl phosphate dissolved in AP buffer (pH 9.0) containing 10% dimethyl formamide overnight at AT. The samples were further washed briefly with MOPS buffer twice, which was replaced with 50% glycerol for examination using a light-field microscope. The embryos from the mBl stage to the Prism stage were stained with PMC surface-specific anti-P4 monoclonal antibody (mAb) in conditioned culture medium without dilution (Shimizu-Nishikawa et al., 1990) to locate the ectodermal AP signal. The mAb was localised with Alexa Fluor-488-tagged anti-mouse IgG antibody (diluted 1:500 in PBST; Molecular Probes Inc., Eugene, OR, USA) by incubation for two hours as described previously (Katow et al., 2004). The anti-P4 mAb-stained WISH samples were examined under an Optiphot epifluorescent microscope (Nikon, Tokyo, Japan). To distinguish the AP signal of WISH from the anti-P4 mAb signal by whole-mount immunohistochemistry (WMIHC), the purple AP-coloured images were artificially converted to red and merged with WMIHC images of anti-P4 mAb using Adobe Photoshop CS5 Extended (ver. 12.0 ×64, Adobe Systems Inc., San Jose, CA, USA).

For WFISH, the embryos from the mBl to the Prism stage were incubated with horseradish peroxidase-conjugated anti-Dig antibody (1:1,000 diluted in blocking buffer) overnight at 4°C. RNA probes were visualised using a Tyramide Signal Amplification system (TSA Plus Cyanine 3/Fluorescein Kits, PerkinElmer, Inc., Waltham, MA, USA) for seven minutes at AT, followed by six washes in MOPS buffer at AT.

The samples were examined using a Micro-Radiance Confocal Laser Scanning Microscope (CLSM; Bio-Rad Microscience, Hemel Hempstead, UK) by counter-staining the nuclei with propidium iodide (SERVA Electrophoresis GmbH, Heidelberg, Germany). Images were analysed with ImageJ (National Institutes of Health), a publicly available image analysis software (http://rsbweb.nih.gov/ij), and Adobe Photoshop CS2 software (ver. 9.02, Adobe Systems Inc., San Jose, CA, USA).

### Immunoblotting and analysis of relative intensity of immunoreaction

To examine the relative intensity of the immunoreaction of GAD, the samples prepared from Ufe, Fe, Morula, sBl, mBl, eG, Prism, 2aPlut and 4aPlut were diluted in sodium dodecyl sulphate-polyacrylamide gel electrophoresis (SDS-PAGE) sample buffer at 1–2 mg ml^−1^, and separated on 10% SDS-PAGE slab gels under reducing conditions, unless described otherwise in the text. The proteins were transferred electrophoretically to nitrocellulose filters (Schleicher and Schuell, Dassel, Germany) at 400 mA for two hours at 4°C. The nitrocellulose blots were blocked with 5% (w/v) skim milk in TBST [50 µmol l^−1^ Tris–HCl (pH 7.0), 0.17 mol l^−1^ NaCl and 0.05% Tween 20] for one hour at AT.

The intensity of the immunoreaction of GAD and tropomyosin that was used as a standard of immunoreaction was analysed using ImageJ Gel plotting software, which is an open-access image analysis software (NIH, USA) (Katow et al., 2013). The experiment was repeated three times with three sets of immunoblots using the samples derived from three pairs of animals, and the difference between mesenchyme blastulae and early gastrulae was analysed by an unpaired *t*-test with QuickCalcs, GraphPad (http://www.graphpad.com/quickcalcs/ttest1.cfm), which is an open-access statistics calculator.

### Whole-mount immunohistochemistry (WMIHC)

The embryos and larvae that had reached the developmental stages described in the text were fixed in 4% PFA for 15–20 minutes, dehydrated in a series of increasing concentrations of ethanol starting from 30% and stored in 70% ethanol at 4°C until use. The samples were hydrated in a decreasing concentration of ethanol to 30% and transferred to 0.1 mol l^−1^ phosphate-buffered saline containing 1% Tween 20 (PBST). Anti-rat GAD_65/67_ rabbit antibody (Enzo Life Sciences International, Plymouth Meeting, PA, USA), which cross-reacts with Hp-Gad (Katow et al., 2013) was diluted 1:500 in PBST. Mouse anti-5HThpr (serotonin receptor) antiserum (Katow et al., 2004) was diluted 1:200 in PBST. The samples were counter-stained for nuclear DNA with 1 µmol l^−1^ propidium iodide (SERVA Electrophoresis GmbH) for two to five minutes during the final washing in PBST. The embryos at the developmental stages described in the text were double-stained with anti-rat GAD_65/67_ rabbit antibody (Enzo Life Sciences International) and an epithelial cell surface-specific anti-Epith-2 mAb diluted 1:10 in PBST (Kanoh et al., 2001; Wakayama et al., 2013) or a skeletogenic PMC-specific anti-SM-50 mouse antibody diluted 1:500 in PBST. All primary antibodies were incubated for 48 hours at 4°C. After washing the samples with PBST three times (10 minutes each), the primary antibodies were detected with Alexa Fluor 488-conjugated goat anti-mouse IgG (H+L) or Alexa Fluor 594-conjugated goat anti-mouse IgG (H+L) antibodies (both from Molecular Probes Inc.) diluted 1:500 or 1:750 in PBST for two hours. The samples were washed three times in PBST as described above, and examined under a CLSM with appropriate optical sections as stated in the figure legends. These images were analysed with ImageJ software (NIH) and Adobe Photoshop CS2 software (ver. 9.02, Adobe Systems Inc.).

The numbers of GADCs in 61 sBl, 50 mBl, 71 eG, 58 mid-gastrulae (mG), 88 30-hpf Prism and 49 33-hpf late Prism (lPrism) stages from two to three different batches were counted on printouts of confocal laser scanning micrographs. The average number of GADCs in the ectoderm and the blastocoel in embryos at each developmental stage was analysed in comparison with those at the previous developmental stage using an unpaired *t*-test with QuickCalcs, GraphPad (http://www.graphpad.com/quickcalcs/ttest1.cfm).

### Bromodeoxyuridine (BrdU) incorporation

Two-dpf 2aPlut were incubated with 2 µg ml^−1^ BrdU (Sigma–Aldrich Co., St Louis, MO, USA) for 24 hours, which is the period during which the number of blastocoelar GADCs increased rapidly, and were fixed with 4% PFA for 15 minutes at AT. The samples were dehydrated from 30% ethanol to 70% ethanol and stored at 4°C until use. After blocking with 5% BSA for one hour, they were incubated with anti-BrdU mAb (Sigma; 1:1,000 diluted in PBST) overnight at 4°C and the mAb was visualized with Alexa Fluor 488-tagged anti-mouse IgG antibody diluted 1:500 in PBST and further incubated with anti-rat GAD_65/67_ rabbit antibody. The antibody was visualized with Alexa Fluor 594-tagged anti-rabbit antibody as described previously (Katow et al., 2004). The number of double-stained GADCs was counted using a total of 195 BrdU-incorporated GADCs in five three-dpf 2aPlut using printouts of the double-stained WMIHC data and analysed by an unpaired *t*-test using QuickCalcs, GraphPad (http://www.graphpad.com/quickcalcs/ttest1.cfm).

### Construction of chimeric embryos

De-jellied Ufe were arranged in rows on plastic dishes that had been coated with 1% protamine sulphate (Wako). After insemination in FSW that contained 1 mmol l^−1^ 3-amino-1*H*-1,2,4-triazole (Wako) to soften the fertilization membrane (Showman and Foerder, 1979), the membrane was removed to enable the manipulation of 16-cell stage embryos. The eggs were microinjected with dextran-rhodamine-B (molecular weight 70,000, neutral; Invitrogen; diluted in distilled water at 2 µg µl^−1^ or 20 µg µl^−1^) using micromanipulators (Narishige, Tokyo, Japan) and Femtojet (Eppendorf Co., Ltd., Tokyo, Japan) or were stained with 10 µg ml^−1^ tetramethylrhodamine isothiocyanate for one hour (Ettensohn and McClay, 1988). At the 16-cell stage, the fluorescein-labelled and -unlabelled embryos were transferred to a glass Petri dish that had been pre-coated with horse serum and filled with Ca^2+^-depleted artificial seawater. They were dissected manually under a dissection microscope using a fine glass needle to separate eight mesomeres. The eight dissected mesomeres from fluorescein-labelled embryos were transferred to fresh Petri dishes that had been pre-coated with 1.2% agar, and were combined with the fluorescein-unlabelled 16-cell stage embryos from which eight mesomeres had been removed as described above. The combined chimeric embryos were incubated in FSW by the method of Amemiya (Amemiya, 1996) until the appropriate developmental stages were reached as described in the text, fixed with 2.5% PFA and processed as described above for WMIHC. The contour of the embryo was obtained by counter-staining with 1 µg ml^−1^ 4′,6-diamidino-2-phenylindole (DAPI) for three minutes during final washing with PBST after secondary antibody application in immunohistochemistry.

## Results

### Spatiotemporal *Hp-gad* transcription pattern by WISH

**Fig. 1.**
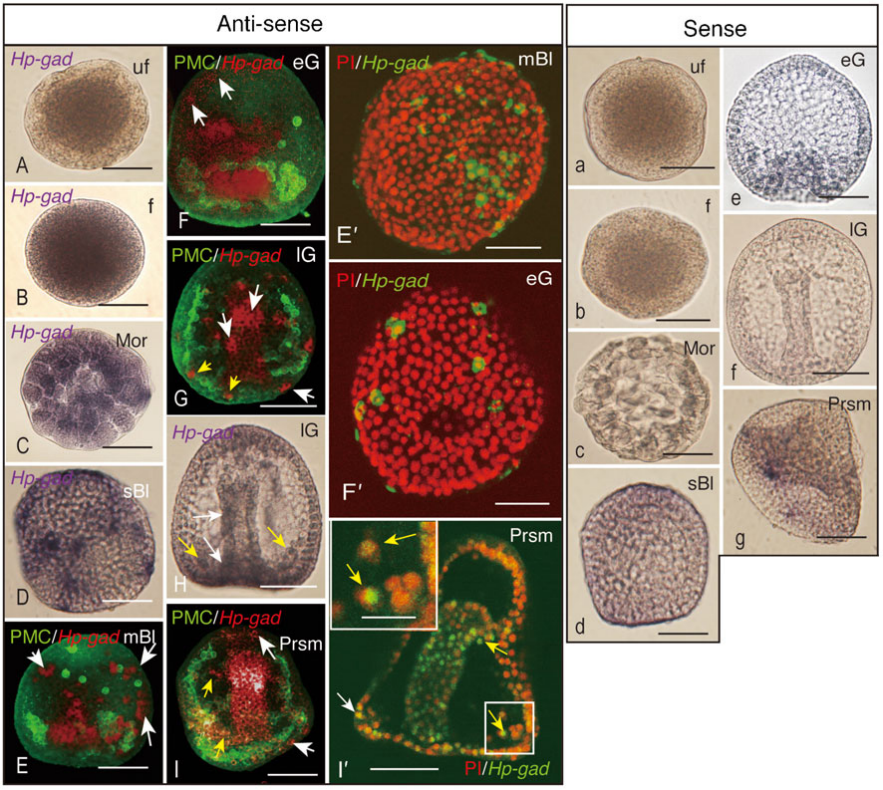
Spatiotemporal pattern of accumulation of *Hp-gad* transcript, determined using whole-mount *in situ* hybridization. (A,a) Unfertilized eggs. (B,b) Fertilized eggs. (C,c) Morulae. (D,d) Swimming blastulae. (E–G,I) Double-stained whole-mount *in situ* hybridization. *Hp-gad* transcripts, red. Primary mesenchyme cells, green. (E) Mesenchyme blastula. (F,F′,e) Early gastrulae. (G,H,f) Late gastrulae. (I,I′,g) Prism larvae. Inset of (I′), higher magnification of the region indicated by a box in (I′). (E′,F′,I′) *Hp-gad*-transcripts, green. Nuclei, red. Arrows, *Hp-gad*-transcribing epithelial cells (white) and blastocoelar cells (yellow). Upper-case letters, anti-sense probe. Lower-case letters, sense probe. Scale bars: 50 µm (A–I′,a–g) and 20 µm (I′ inset).

A low level of *Hp-gad* transcript was detected in the ooplasm of Ufe (Fig. 1A,a) and moderate accumulation of *Hp-gad* transcript was detected in Fe (Fig. 1B,b). In Morulae, the transcripts accumulated throughout the blastomeres (Fig. 1C,c). By the sBl stage, transcript accumulation was particularly apparent in clusters that each comprises one to three cells and were distributed throughout the entire ectoderm (Fig. 1D,d), which continued to the mBl stage (Fig. 1E,E′) and the eG stage (Fig. 1F,F′,e). In eG, GADCs were seen apparent in the blastocoel (Fig. 1F, arrows). Considerable accumulation of *Hp-gad* transcripts was noted in the gut epithelium (Fig. 1G, arrow, Fig. 1H, white arrows); this was also apparent in blastocoelar cells (Fig. 1G,H, yellow arrows) in lG. The elevated accumulation of *Hp-gad* transcripts was detected at the Prism stage, which was associated with an increased number of GADCs in the gut epithelium (Fig. 1I,I′). Thus, *Hp-gad* transcripts accumulated throughout the ectodermal region and, except for in PMCs (Fig. 1E–G,I), in some of the blastocoelar cells in Prism (Fig. 1I′, inset). This implicates that PMCs are not the sole cell type in the blastocoel of eG, which will be discussed further later in this report. The present observation traced the onset of *Hp-gad* transcription back to Ufe from at around the Prism stage, which was reported previously based on the RT-PCR analysis (Katow et al., 2013).

### Spatiotemporal expression pattern of Hp-Gad

**Fig. 2.**
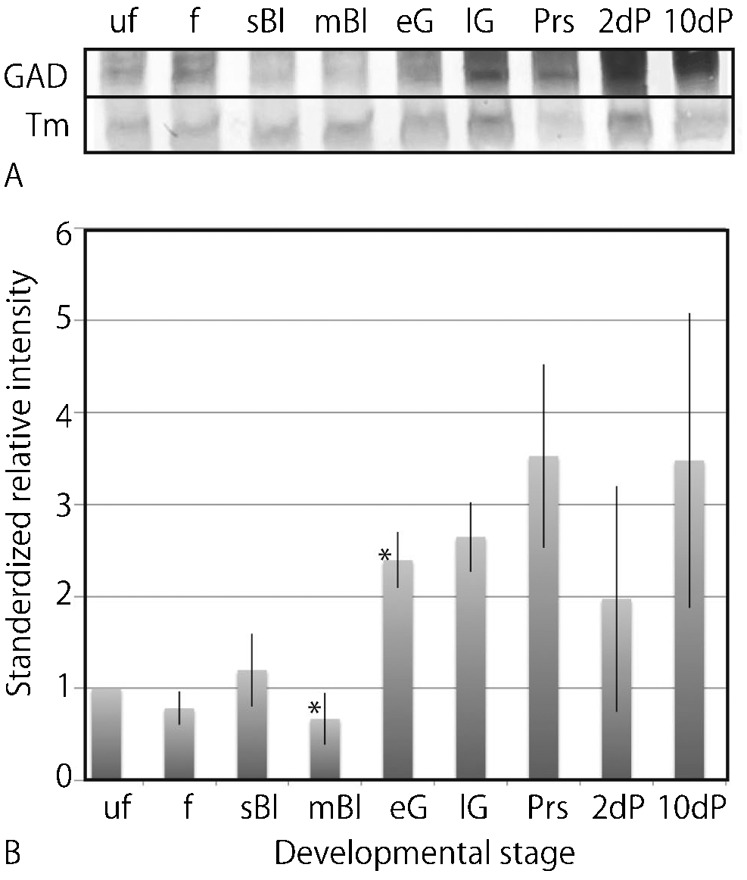
Temporal changes in the expression of Hp-Gad during development, as determined by immunoblotting. (A) Immunoblotting pattern of Hp-Gad (GAD). Uf, unfertilized eggs. f, fertilized eggs. sBl, swimming blastulae. mBl, mesenchyme blastulae. eG, early gastrulae. lG, late gastrulae. Prs, prism larvae. 2 dp, two-day post-fertilization (-dpf) two-arm plutei. 10 dp, 10-dpf four-arm plutei. Tm, sea urchin tropomyosin. (B) Relative intensity of immunoreaction of Hp-Gad during development. Abscissa, developmental stage with the same abbreviations as in (A). Vertical bars, s.d. Asterisks indicate significant differences determined using unpaired *t*-test (*P*<0.005).

Consistent with the above pattern of *Hp-gad* transcript accumulation, Hp-Gad was detected during the same period of the development that started from in Ufe and continued to as late as the 10-dpf 4aPlut stage by immunoblotting. During that period, the *M_r_* of Hp-Gad was consistently predicted to be approximately 80 kDa region (Fig. 2A). However, the relative intensity of the immunoreaction (RIM) indicated dynamic changes. Until the mBl stage, the RIM remained low, but increased considerably at and after the eG stage from the previous mBl stage (*P*<0.005; Fig. 2B). The RIM remained at a similar level until the 10-dpf 4aPlut stage. Considering the present WISH results, the significant RIM increase occurred in association with gastrulation, when the *Hp-gad* transcript accumulation intensified at the archenteron and in the blastocoelar GADCs (Fig. 1).

**Fig. 3.**
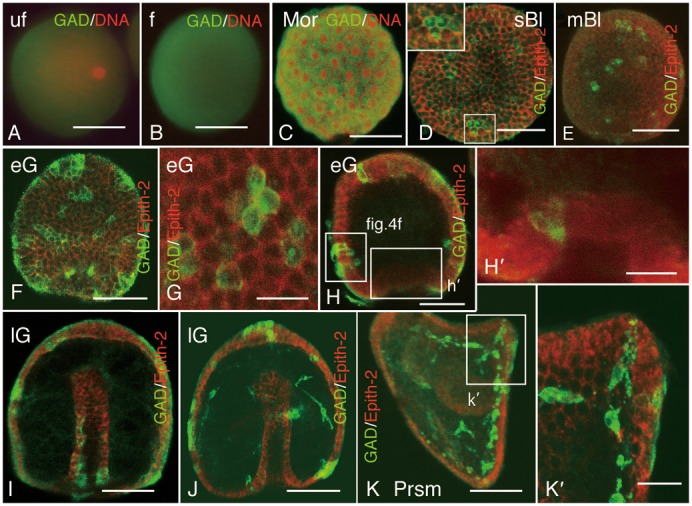
Spatiotemporal expression pattern of Hp-Gad (green) during development, determined using double-stained whole-mount immunohistochemistry with nuclei (red; A–C) or Epith-2 (red; D–K′). (A) Unfertilized eggs. (B) Fertilized eggs. (C) Morula. (D) Swimming blastula. Inset, higher magnification of boxed area. (E) Mesenchyme blastula. (F) Early gastrula. (G) Surface of early gastrula. (H) Optical section of early gastrula. (H′) Higher magnification of an optical section at the blastopore region indicated by a box (h′) in (H). (I) Optical section of late gastrula. (J) Optical section of late gastrula. (K) Optical section of prism larva (Prsm). (K′) Higher magnification of a box (k′) in (K). Scale bars: 10 µm (G), 20 µm (H′,K′) and 50 µm (A–F,H–K).

**Fig. 4.**
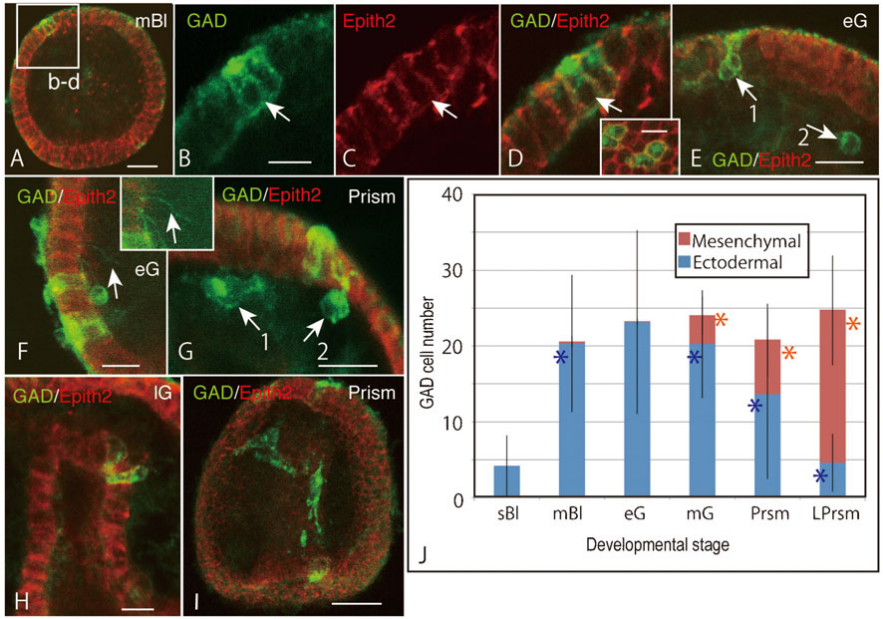
Ingression of GAD-expressing cells (GADCs; green) and an increasing number of blastocoelar GADCs during early development, as indicated by double-stained whole-mount immunohistochemistry with Hp-Gad (green) and Epith-2 (red). (A) Optical section of mesenchyme blastula. Box (b–d) is shown with higher magnification from (B) to (D). (B) Enlarged ectodermal GADCs indicated by box (b) in (A). Arrow, GADC. (C) Epith-2 on the surface of GADCs (arrow) of (B). (D) Merged image between (B) and (C). Inset, Optical sagittal section of the ectoderm shows Hp-Gad-positive cytoplasmic dot and the plasma membrane. (E) Animal ectoderm of early gastrula. Pear-shaped GADCs (arrow 1) in animal ectoderm and round GADC in the blastocoel (arrow 2). (F) Higher magnification of vegetal ectoderm of early gastrula of Fig. 3H. GADCs in the ectoderm and basipetal extension of fine filopodia (arrow). Inset, high-contrast image of filopodia (arrow) indicated by an arrow in the mainframe. (G) Prism larva. GADCs on the basal surface of ectoderm (arrow 1) and that showing transient morphology of ingression (arrow 2). (H) Late gastrula. GADCs in the wall of archenteron near the tip region. (I) Prism larva. Spindle-shaped GADCs in the blastocoel. (J) The proportions of GADCs in the ectoderm (blue columns) and in the blastocoel (red columns). Scale bars: 30 µm (A,I), 10 µm (B,D inset,H), 15 µm (E–G). Error bars, s.d. *, statistically significant difference from the one to the immediate left by unpaired *t*-test (*P*<0.0001).

The spatial localization of Hp-Gad in eggs before and after fertilization remained similar and evenly distributed throughout the ooplasm (Fig. 3A,B). During the Morula stage, although Hp-Gad was not associated with sub-cellular features, the immunoreactivity of Hp-Gad intensified in the perikaryon with a clear deficient area near the plasma membrane in the entire ectodermal region (Fig. 3C). By the sBl stage, the number of GADCs had decreased considerably in the ectoderm in association with convergence to clumps of ectodermal GADCs. In each GADC, Hp-Gad was expressed being associated with a cytoplasm dot and the area near the plasma membrane (Fig. 3D, inset), which remained similar to the mBl stage (Fig. 3E). In eG, the number of ectodermal clumps of GADCs increased considerably all over the embryo (Fig. 3F), which apparently correlated with the aforementioned increase in the RIM that was determined by immunoblotting. Although the sub-cellular localization of Hp-Gad remained similar to that in the younger embryos starting from the sBl stage, the immunoreaction of Hp-Gad in the cytoplasmic dot weakened during the course of subsequent development, whereas the signal near the plasma membrane was intensified (Fig. 3G). Throughout the ectodermal area of eG, the shape of GADCs elongated in apicobasal polarity (Fig. 3H, Fig. 4F). The resemblance between this change and a similar change in cell shape during PMCs ingression (Katow and Solursh, 1980) suggested the occurrence of similar delamination of GADCs from the ectoderm. The epithelium of gut also formed GADCs (Fig. 3H′), as was also shown by the present WISH (Fig. 1G,H). By the lG stage, GADCs were seen throughout gut epithelium (Fig. 3I) and the blastocoel (Fig. 3J). At the Prism stage, blastocoelar GADCs constituted a network (Fig. 3K) and some of them infiltrated into the ectoderm at the tips of the early anal arms (Fig. 3K′).

### Ingression of ectodermal GADCs

To examine whether the blastocoelar GADCs are derived from the ectoderm by ingression, the histological localization of GADCs was examined in detail from the mBl stage to the Prism stage. In mBl, the ectodermal GADCs were first noticed with a GAD signal associated with a cytoplasmic dot on the apical side and along the plasma membrane (Fig. 4A,B,D, arrow, Fig. 4D, inset). These GADCs also expressed the epithelial cell surface-specific Epith-2 protein (Fig. 4C,D, arrow). Thus, GADCs were a population of ectodermal cells. Only a few blastocoelar GADCs were seen at this stage. During the eG stage, some of the ectodermal GADCs appeared to show the potential for ingression into the blastocoel (Fig. 4E, arrow 1, Fig. 4G, arrow 2, Fig. 4H). There were also the blastocoelar GADCs on the basal side of the ectoderm (Fig. 4E, arrow 2). Some of the ectodermal GADCs formed basipetally extended very thin and long filopodia (Fig. 4F, inset, arrow). In Prism, the number of blastocoelar GADCs increased (Fig. 4G, arrow 1). Some GADCs maintained a part of their cell body in the ectoderm (Fig. 4G, arrow 2). GADCs for which some of the cell body protruded toward the blastocoel were also detected in the gut epithelium in lG (Fig. 4H). In Prism, the GADCs in the ectoderm apparently decreased in number, whereas those that showed a spindle shape apparently increased and spread out in the blastocoel (Fig. 4I). Thus, GADCs that appeared first in the ectoderm in mBl had apparently shifted into the blastocoel by the Prism stage.

Consistent with the findings mentioned above, the quantitative analysis also indicated that the total number of GADCs increased significantly from the sBl stage to the eG stage, with a very small proportion of blastocoelar cells (Fig. 4J). Nonetheless, whereas the majority of GADCs were in the ectoderm until the eG stage, the proportion of blastocoelar GADCs increased noticeably in mG (about 20%). The blastocoelar GADCs increased steadily during the subsequent developmental period to reverse the proportion to reach about 80% of total GADCs at the lPrism stage. This occurred without a notable increase in the total number of GADCs, which reached a plateau at and after the mG stage. Thus, the observed change in the composition of GADCs between the ectoderm and the blastocoel suggests at least the following three possibilities; (1) active proliferation of a small proportion of ingressed blastocoelar GADCs associated with an inversely proportional decrease in the number of ectodermal GADCs following termination of *Hp-gad* expression; (2) inversely proportional shifting of Hp-Gad-expressing sites from the ectodermal cells, without a shift of the location of GADCs; and (3) active ingression of ectodermal GADCs to the blastocoel.

**Fig. 5.**
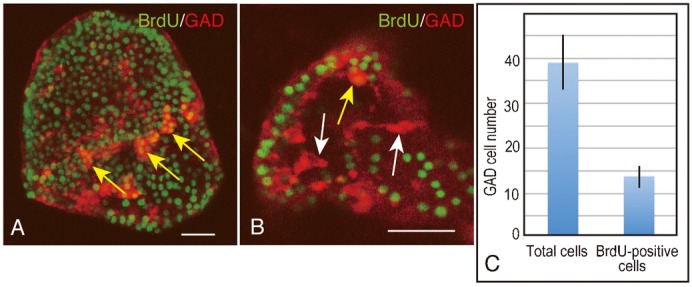
Proportion of bromodeoxyuridine (BrdU; green)-incorporated Hp-Gad-expressing cells (GADCs; red) in two-dpf two-arm (2aPlut) plutei analyzed by double-stained whole-mount immunohistochemistry. (A) Stacked image of 2aPlut. Arrows, BrdU-positive blastocoelar GADCs. (B) Optical section of 2aPlut. Yellow arrow, BrdU-positive GADCs. White arrows, BrdU-negative blastocoelar GADCs. Scale bars: 20 µm. (C) The proportion of BrdU-incorporated GADCs in 2aPlut is significantly smaller than that of total GADCs (*P*<0.0005), as determined by an unpaired *t*-test. Error bars, s.d.

The first hypothesis was tested in terms of whether blastocoelar GADCs proliferate by using the detection of BrdU, which was applied during the period of active increase in the number of blastocoelar GADCs; however, the obtained result was an insubstantial increase in the proportion of BrdU-incorporated blastocoelar GADCs (Fig. 5A,B). They shared only about 30% of total GADCs in 2aPlut (Fig. 5C). Thus, the first hypothesis does not fully explain how the 80% of GADCs were supplied in 2aPlut.

The second and third hypotheses require the absence (for the second hypothesis) or presence (for the third hypothesis) of ectodermal descendants among blastocoelar GADCs. Although the chronological increase of GADCs observed in the present study was associated with the transient morphology of ingression (Fig. 4E,G,H), this was slightly inconsistent with the second hypothesis, so the latter two hypotheses could be tested by an experiment to detect blastocoelar GADCs with ectodermal cell markers. However, according to our understanding of the sea urchin lineage, the vegetal ectoderm produces two blastocoelar mesenchyme cell types: PMCs and SMCs (Hörstadius, 1939; Cameron et al., 1987; Davidson et al., 1998). Thus, unless we confirm that the blastocoelar GADCs are not descendants of SMCs, detecting the presence or absence of blastocoelar GADCs with an ectodermal marker alone is insufficient to support either hypothesis. The WMIHC results generated in the present study suggested the involvement of the entire ectodermal regions in supplying the blastocoelar GADCs. These regions include the animal ectoderm, which is derived from the mesomeres of the 16-cell stage embryo, but does not produce the mesenchyme (Hörstadius, 1939; Cameron et al., 1987; Davidson et al., 1998). Despite this, if certain blastocoelar GADCs indicate the animal ectoderm-specific marker, SMC will be excluded from the cell lineage of blastocoelar GADCs.

### Ontogenetic analysis of the origin of GADC in chimeric embryos

**Fig. 6.**
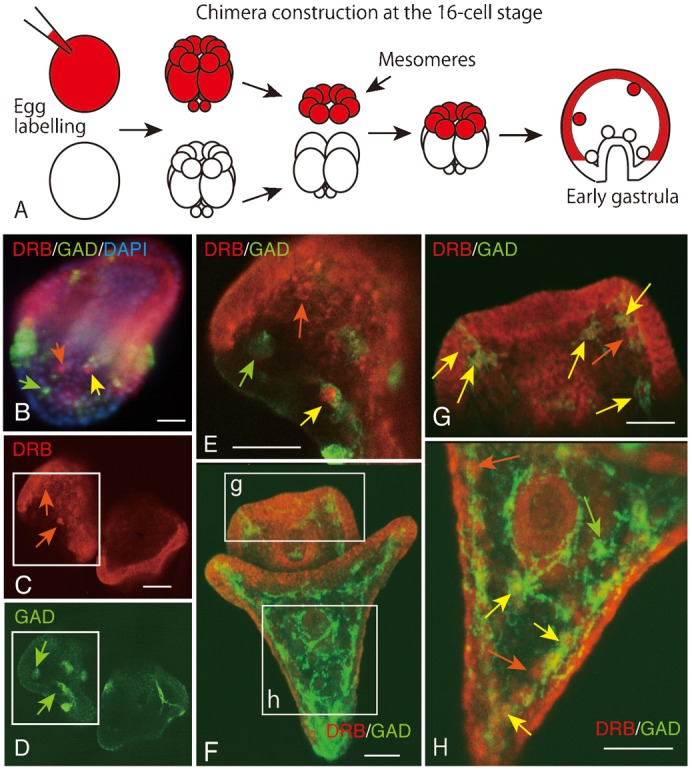
Schematic representation of the chimeric 16-cell stage embryo (A) and double-stained whole-mount immunohistochemistry with Hp-Gad (green) and fluorescein (red) of chimeric prism larva (B–E) and two-dpf two-arm plutei (F–H). (A) Schematic of chimera construction between fluorescein-stained mesomeres (red) and unlabelled macro- and micromeres. (B) Fluorescence micrograph of triple-stained prism larva. Red, animal ectoderm. Blue, 4′,6-diamidino-2-phenylindole. Green, Hp-Gad. Green arrow, GADCs. Red arrow, dextran-rhodamine-B-stained cells. Yellow arrow, cells that were stained with Hp-Gad and dextran-rhodamine-B. (C) Four-micrometer-thick optical section of dextran-rhodamine-B-labelled animal ectoderm in prism larva. (D) GADCs in the same larva as in (C). (E) Merged image of (C) and (D). Yellow arrow, mesomere-derived GADC. Green arrow, vegetal ectoderm-derived GADC. Red arrow, animal ectoderm-derived non-GADCs. Vegetal ectoderm is not stained with dextran-rhodamine-B. (F) Ninety-five-micrometer-thick stacked image of two-day post-fertilization two-arm pluteus larva. Boxes (g) and (h) are highly magnified in (G) and (H). (G) Four-micrometer-thick optical section of oral lobe are indicated by the box (g) in (F). Yellow arrows, TRITC-positive GADCs. Orange arrow, TRITC-positive non-GADCs. (H) Forty-micrometer-thick optical section of posterior trunk region indicated by the box (h) in (F). Yellow arrows, TRITC-positive GADCs. Green arrow, TRITC-negative GADC. Orange arrows, GAD-negative TRITC-positive cells. Scale bars: 30 µm.

To examine the presence of mesomere-derived blastocoelar mesenchyme, we constructed chimeric embryos by combining mesomeres from red fluorescein-stained 16-cell stage embryos and the vegetal blastomeres: the macromeres and the micromeres from the embryos of the same developmental stage that were not stained by fluorescein (Fig. 6A). The concept behind this experiment is that, in the chimeric embryo, all blastocoelar GADCs that are derived from mesomeres are fluorescein-positive. This distinguishes them from the GADCs from other cell lineages, such as SMC descendants.

The present chimeric embryos reached the Prism stage with red fluorescein-labelled animal ectoderm, green GADCs in the ectoderm and the blastocoel, and non-fluorescein-stained vegetal ectoderm that was stained blue by DAPI (Fig. 6B). Examination of optical sections of chimeric embryos using a CLSM showed red fluorescein-positive animal ectoderm and blastocoelar mesenchyme cells (Fig. 6C, arrows), which indicated the presence of mesomere-derived mesenchyme cells. In these same chimeric embryos, GADCs were also detected in the ectoderm and the blastocoel (Fig. 6D, arrows), which was consistent with the present WMIHC data for intact embryos obtained during the current study (Fig. 4). The merged image of the above two detected red-fluorescein-positive GADCs (Fig. 6E, yellow arrow) among the fluorescein-negative GADCs (Fig. 6E, green arrow) and the fluorescein-positive cells in the blastocoel that are not GADCs (Fig. 6E, red arrow). This observation confirmed the presence of the animal ectoderm-derived blastocoelar mesenchyme and hence the presence of the mesomere-derived blastocoelar GADCs.

In two-dpf 2aPlut, blastocoelar GADCs constituted a cellular network (Katow et al., 2013) (Fig. 6F). Closer examination at a higher magnification depicted a conserved ontogenetically heterogeneous population of blastocoelar GADCs from the younger embryos. The mesomere-derived GADCs (Fig. 6G, yellow arrows) were seen among vegetal ectoderm-derived non-GADCs (Fig. 6G, red arrow) in the oral lobe blastocoel. The vegetal ectoderm-derived GADCs (Fig. 6H, green arrow) and mesomere-derived GADCs (Fig. 6H, yellow arrows) were seen among the vegetal ectoderm-derived non-GADCs (Fig. 6H, red arrows) in the posterior trunk blastocoel.

**Fig. 7.**
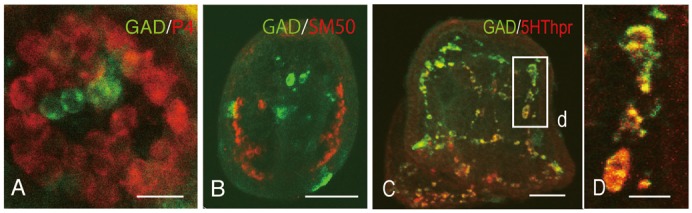
Heterogeneous blastocoelar cells in early gastrulae and expression of multiple proteins in a blastocoelar cell in plutei analysed by double-stained whole-mount immunohistochemistry. (A) Early gastrula. Green, glutamate decarboxylase (GAD)-expressing cell (GADC), Red, primary mesenchyme cell. (B) Late gastrula. Green, GADCs. Red, SM50-expressing cell. (C) Prism larva. Green, GAD. Red, serotonin receptor (5HThpr). (D) High magnification of cells indicated by the box (d) in (C). Scale bars: 20 µm (A), 40 µm (B), 25 µm (C), 10 µm (D).

Ontogenetically heterogeneous blastocoelar mesenchyme, namely, GADCs from the ectoderm and PMCs from the large micromeres, was detected in eG, in which SMC is not yet formed, and yet a group of GADCs constituted a clump of cells with PMCs near the blastopore (Fig. 7A). However, these GADCs never participated in skeletogenesis according to the observation of a clear separation of SM50-positive PMCs from GADCs in lG (Fig. 7B). Thus, a heterogeneous blastocoelar cell population occurred even at and after the eG stage in which no non-PMCs are present (Hörstadius, 1939).

The blastocoel comprises several types of mesenchyme cells, including a major network of SMC-derived SRCs (Katow et al., 2004; Katow et al., 2007) (Fig. 6C). Some of these SRCs expressed GAD. Interestingly, the serotonin receptor protein and GAD were expressed in the similar sub-cellular compartments or at different intensities in the same cells (Fig. 7C,D), which is consistent with the present observation that GADCs were derived from all of the blastomeres.

## Discussion

### Mesomere-derived mesenchymal GADCs

In sea urchin embryos, SMCs are the only exclusively multipotent mesenchymal cells. The ontogenetic origin of SMCs during normal development has been well documented (Cameron et al., 1987). Under experimental conditions, SMCs display a limited capacity for conversion to skeletogenic cells, which is a property that they share with PMCs, under experimental condition (Ettensohn and McClay, 1988). Given that SMCs are descendants of the macromeres (Cameron et al., 1987), the SMC-converted skeletogenic cells are associated with the inter-blastomere conversion between descendants of macromeres and micromeres (Cameron et al., 1987). On the other hand, the GADCs in the blastocoel included those from the animal ectoderm, descendants of the mesomeres, and those from the vegetal ectoderm, descendants of the macro- and micromeres. Thus, the GADCs are the descendants of all three blastomeres. This feature of a multi-ancestral ontogenetic origin of pluripotent mesenchyme is reminiscent of the recent concept of a mesenchyme stem cell (MSC) lineage in vertebrates (Schipani and Kronenberg, 2009 (http://www.ncbi.nlm.nih.gov/books/NBK27056)). The present observation implies the presence of a new mesenchymal cell group, tertiary mesenchyme cells (TMCs).

Classical knowledge of cell lineages in sea urchin came from a series of experiments using fluoresceinated dextran that was microinjected into individual blastomeres of embryos of the sea urchin, *S. purpuratus*, at the eight-cell stage, with analysis in three-dpf 2aPlut under a fluorescence microscope using whole-mount samples and paraffin sections. In that study, the failure to detect fluorescein-labelled animal blastomere (Na, NL and No)-derived cells in the mesenchyme (Cameron et al., 1987) contradicts the observations made in the present study. In view of the very similar, if not identical, developmental features and genomic structures between *H. pulcherrimus* and *S. purpuratus*, the possible cause of this discordance might be related to differences in the experimental techniques used in these studies. Even in our present analysis of the triple-stained WMIHC with a fluorescence microscope, the exact location of apparent blastocoelar cells was slightly unclear due to the rather poor resolution (Fig. 6B). However, the CLSM used in the present study enabled localization of the exact position of signal-positive cells in the blastocoel with superb resolution (Fig. 6C–H).

The present observation of the mesomere-derived TMC might help to improve our understanding of the connections in the gene regulatory network that controls sea urchin development. This includes the phenotypic flexibility of the TMC in the blastocoel, as exemplified by simultaneous expression of GAD and the serotonin receptor protein in the same cell. The simultaneous expression of these two proteins in single blastocoelar GADCs will be further discussed later in this section.

### GADC formation in and ingression from the ectoderm

In plutei, GADCs also express GABA (Katow et al., 2013), as has been previously reported in vertebrates (Mueller et al., 2006). Immunohistochemistry indicated that the earliest GABA-expressing cells were detected in the blastocoel at around the Prism stage (Katow et al., 2013), whereas the present WISH/WFISH analysis detected *Hp-gad*-transcribing cells earlier at the sBl stage, which resembles the preceding transcription of *GAD* and delayed detection of GABA in zebrafish brain (Mueller et al., 2008). This time lag suggests that enzyme expression does not result in immediate synthesis of the neurotransmitter. This might result from delayed activation of the enzyme, as has been reported in developing rat brain, for which northern blotting indicated that enzyme activation takes about three weeks after the protein expression at embryonic day 15; likewise the onset of accumulation of *Gad* transcripts occurs more than two weeks earlier than the appearance of GAD protein (Bond et al., 1988). Regarding the occurrence of *Hp-gad* transcript accumulation during cleavage period when the embryos do not yet differentiate the GABAergic nervous system, GABA may be involved in non-neural functions, such as morphogenesis including cleavage (Buznikov et al., 1964; Moiseiwitsch, 2000; Wee and Zimmerman, 1983) and should be addressed in future studies in sea urchin embryos.

The expression in the ectodermal placode of *Gad1*, which encodes the 67-kDa isoform of GAD in mouse embryo, is associated with the expression in the MSC population of the tailbud and in the pharyngeal endoderm and mesenchyme (Maddox and Condie, 2001). Above all, like the present Hp-Gad expression in sea urchin, *Gad1* is expressed in several structures that are derived from each of the three germ layers of the embryo. The sites of *Gad1* expression are the tissues that emit signals required for patterning and differentiation (Maddox and Condie, 2001), which include tissues that express LIM-homeobox genes. The gene family that encodes LIM-homeobox proteins is involved in differentiation and neural patterning in diverse organisms (Hobert and Westphal, 2000). A number of preplate GABAergic cells in human cerebral cortex co-express *Dlx* (Stühmer et al., 2002; Rakic and Zecevic, 2003). *Dlx1/2* activates GAD/67, the rate-limiting enzyme for GABAergic synthesis (Stühmer et al., 2002), by interaction with *Asc1/Mash1* in the telencephalon (Evans et al., 2012). However, in sea urchin embryos, the apparent spatial pattern of accumulation of *Sp-dlx* transcripts determined using *in situ* hybridization (Howard-Ashby et al., 2006) does not overlap with the spatial pattern of accumulation of *Hp-gad* transcripts. Thus, *dlx* may not participate in the regulation of *Hp-gad* activation. On the other hand, the spatiotemporal expression pattern of *Sp-lhx2* (Howard-Ashby et al., 2006) is similar to that of *Hp-gad*/Hp-Gad in the ectoderm. Whether *Lhx2* regulates GADC differentiation remains to be examined. Regarding GADC differentiation in mouse brain under the regulation that involves *megane* (Guimera et al., 2006), the contribution of the βHLH transcription factor should also be considered in sea urchin embryos.

Like echinochrome synthetase expression in pigment cell precursors before the onset of ingression in the blastula stage in *S. purpuratus* (Calestani and Rogers, 2010) and cell surface-specific P4 expression in the ectoderm before ingression in *H. pulcherrimus* (Shimizu-Nishikawa et al., 1990; Katow, 1991), the expression of Hp-Gad occurred before ingression from the ectoderm. These two mesenchyme cells, SMCs and PMCs, internalize Epith-2, an epithelial cell-surface protein, during ingression by endocytosis (Wakayama et al., 2013). The present GADCs also lost Epith-2 during ingression. Thus, ingression of PMCs, SMCs and TMCs proceeds by fairly similar processes.

### Dual expression of neural transmitters

The dual expression of GAD and serotonin receptor in a single blastocoelar cell, as observed in the present study, is not unique to sea urchin, but instead is widely recognized in vertebrates, such as in stage 42–48 *Xenopus* tadpole (Huang and Moody, 1998), the prefrontal cortex of rat and primates (Jakab and Goldman-Rakic, 1998; Williams et al., 2002; Yan, 2002; Santana et al., 2004) and mouse spinal superficial dorsal horn (Fukushima et al., 2009). Whether the dual expression in the same blastocoelar TMC is a temporary phenotype at the Prism stage or the terminal phenotype that lasts through the larval stages in sea urchin or throughout the adulthood remains to be elucidated. However, the dual expression suggests that serotonin receptors contribute to the complex regulation of GABAergic inhibitory transmission in the prefrontal cortex (Yan, 2002) by modulating excitatory and inhibitory effects mediated by glutamate and GABA, respectively (Ciranna, 2006), or by regulating GABA_A_ receptor-mediated inhibitory synaptic transmission in the superficial dorsal horn (Fukushima et al., 2009). Thus, the present observation provides a structural basis for our previous observations that embryonic and larval swimming is regulated by all three classical NSs through crosstalk among them (Yaguchi and Katow, 2003; Katow et al., 2004; Katow et al., 2007; Katow et al., 2010; Katow et al., 2013). In particular, at least serotonergic and GABAergic NSs detected in the blastocoelar cells may constitute a crosstalk circuit in the blastocoelar cell network from around the Prism stage in which both nervous RSs appear (Katow et al., 2013). There will also be neurotropic interaction that is necessary to maintain blastocoelar SRC (Katow et al., 2007); the present observation suggests that this also involves the regulation of GAD-mediated GABA synthesis by serotonin. The present results leave open the question of whether cells that simultaneously express GAD and the serotonin receptor also include SMCs; this remains to be addressed.
